# 2114. Invasive fusariosis in the era of mold active azoles and molecular targeted chemotherapy: Increasing incidence and lack of improved outcomes

**DOI:** 10.1093/ofid/ofac492.1735

**Published:** 2022-12-15

**Authors:** Takahiro Matsuo, Sebastian Wurster, Ying Jiang, Koji Sasaki, Jeffrey J Tarrand, Russell E Lewis, Dimiitrios P Kontoyiannis

**Affiliations:** The University of Texas MD Anderson Cancer Center, Houston, Texas; The University of Texas MD Anderson Cancer Center, Houston, Texas; The University of Texas MD Anderson Cancer Center, Houston, Texas; The University of Texas MD Anderson Cancer Center, Houston, Texas; The University of Texas MD Anderson Cancer Center, Houston, Texas; University of Bologna, Bologna, Emilia-Romagna, Italy; The University of Texas MD Anderson Cancer Center, Houston, Texas

## Abstract

**Background:**

Invasive fusariosis (IF) is an uncommon opportunistic mold infection primarily affecting patients (pts) with leukemia and allogeneic hematopoietic cell transplant (HSCT). Historically, IF had poor outcome when there is lack of recovery from immunosuppression. However, IF incidence and outcome in the in the era of new mold-active triazoles and leukemia regimens that incorporate molecularly targeted drugs is unknown.

**Methods:**

We retrospectively studied the incidence, risk factors, clinical features, and outcome of microbiologically documented IF (revised EORTC/MSG criteria) in pts with leukemia at MD Anderson Cancer Center in the last 12 years (June 2009-October 2021). The independent risk factors for 42-day (d) mortality from IF diagnosis were assessed by a binary multivariable logistic regression model. Annual incidence density (1998-2021) was estimated using Poisson regression analysis.

**Results:**

Among 140 IF pts (114 proven, 26 probable), 100 (71%) had pneumonia, 88 (63%) disseminated infection, and 32 (23%) sinusitis, with no apparent changes in relative frequency over the years. 124 pts (89%) had neutropenia (ANC < 500) at IF diagnosis, 118 (84%) had relapsed/refractory (R/R) leukemia, and 43 (31%) had prior HSCT. 19% (18/97 pts) of IF pts had positive serum galactomannan and 55% had coinfections. In the last 5 years of the study, 45/58 IF cases (78%) were breakthrough infections to mold active triazoles (12 voriconazole, 25 posaconazole, 8 isavuconazole). 74 (53%) out of the 140 IF pts died by d42. Only 6 pts (9%) had neutrophil recovery at time of death compared to 47 (64%) who survived until day 42 (P < 0.001). Neutrophil recovery (aOR 0.04, 95% CI 0.01-0.14), SOFA score (aOR 1.91 per point, 95% CI 1.47-2.50), and pneumonia (aOR 3.28, 95% CI 1.11-9.70) were independent predictors of 42-d mortality. Since 1998, the incidence density of IF continuously increased (P = 0.006) at an annual ratio of 1.05 (95% CI 1.02-1.07, **Figure 1**).

**Figure 1:**
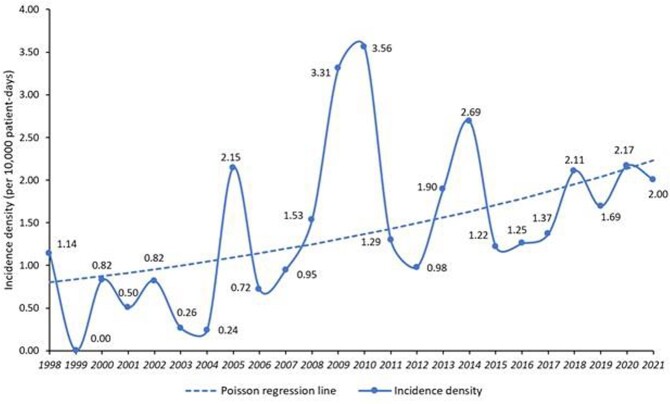
Incidence density of invasive fusariosis (per 100,000 patient-days) in leukemia patients between 1998 and 2021

**Conclusion:**

Over the past 23 years, IF incidence has been increasing. IF is predominantly seen in pts with R/R acute leukemia and increasingly seen as a breakthrough infection to mold-active triazoles. Even in contemporary pt cohorts, IF has high mortality in the setting of persistent myelosuppression.

**Disclosures:**

**Russell E. Lewis, PharmD**, Air: Advisor/Consultant|Cidara: Advisor/Consultant|F2G: Advisor/Consultant|Gilead: Advisor/Consultant|Merck & Co, Inc: Grant/Research Support|Synexis: Advisor/Consultant **Dimiitrios P. Kontoyiannis, MD, ScD, PhD (hon)**, AbbVie: Advisor/Consultant|Astellas Pharma: Advisor/Consultant|Astellas Pharma: Grant/Research Support|Astellas Pharma: Honoraria|Cidara Therapeutics: Advisor/Consultant|Gilead Sciences: Advisor/Consultant|Gilead Sciences: Grant/Research Support|Gilead Sciences: Honoraria|Merck: Advisor/Consultant.

